# Improving assessment of acute obstetric patients – introducing a Swedish obstetric triage system

**DOI:** 10.1186/s12913-021-07210-9

**Published:** 2021-11-06

**Authors:** Linnéa Lindroos, Radha Korsoski, Marie Ordéus Öhman, Helen Elden, Ove Karlsson, Verena Sengpiel

**Affiliations:** 1 Department of Obstetrics, Sahlgrenska University Hospital/Östra, Diagnosvägen 15, Paviljong 7b, 416 50 Gothenburg, Sweden; 2 Department of Obstetrics and Gynecology, Institution of Clinical Sciences, Sahlgrenska Academy, University of Gothenburg, Gothenburg, Sweden; 3 Institution of Health and Care Sciences, University of Gothenburg, Gothenburg, Sweden; 4 NU Hospital Group, Trollhättan, Sweden; 5 Department of Anesthesiology and Intensive Care, Institution of Clinical Sciences, Sahlgrenska Academy, University of Gothenburg, Gothenburg, Sweden

**Keywords:** Triage, Obstetrics, Pregnancy, Emergency medicine, Quality improvement

## Abstract

**Background:**

Failure to identify severely ill obstetric patients seeking acute care, and hence delaying treatment, can lead to maternal morbidity and mortality. Triage is the prioritization of patients seeking emergency care, based on clinical decision-making tools assessing medical urgency. While triage has been applied in general emergency medicine for 30 years, there are only a few obstetric triage systems (OTS) and obstetric triage has hitherto been unknown in Sweden. Obstetric triage is more complex than general triage since both mother and fetus require assessment, and pregnancy-related physiological changes must be taken into account. This paper aims to describe the development and an initial evaluation of the first OTS in Sweden.

**Methods:**

A multidisciplinary team surveyed reasons to seek acute obstetric care and the current patient flow at the largest obstetric unit in Scandinavia, Sahlgrenska University Hospital, Gothenburg, Sweden, with about 10,000 deliveries/year. A semi-structured literature review on obstetric triage was undertaken. Based on the survey and the literature review the first Swedish OTS was developed and implemented. Patient satisfaction was followed by electronical questionnaires. Initial validity evaluation was performed, defined by the system’s ability to identify patients with need for hospital admission, stratified by acuity level.

**Results:**

The Gothenburg Obstetrical Triage System (GOTS) addresses the patient to one of five acuity levels based on both vital signs and 14 chief complaint algorithms. It entails recommendations for initial procedures of care as well as an acuity form for documentation. Initial evaluation of the system indicates good correlation between need for admission and acuity level. The implementation has provided the staff with an improved medical overview of the patients and patient flow and enabled the unit to monitor emergency care in a structured way. Implementation came along with increased patient and staff satisfaction.

**Conclusion:**

The GOTS is the first OTS developed in and for Sweden and implementation has improved management of obstetric patients seeking acute care. Patients are now prioritized according to level of acuity and the time to assessment and treatment of severely ill patients can be structurally evaluated. Both patients and staff express improved satisfaction with obstetric triage.

**Supplementary Information:**

The online version contains supplementary material available at 10.1186/s12913-021-07210-9.

## Background

For non-obstetric patients seeking acute care at an emergency department (ED), triage is performed at first contact with medical staff in order to determine acuity level and hence prioritize in accordance with clinical urgency. There are myriad triage systems and triage has been the gold standard in general emergency care for more than 50 years [1]. In Sweden, the predominant non-obstetric triage system is the Rapid Emergency Triage and Treatment System (RETTS) [2, 3]. Triage has been shown to reduce the total length of stay (LOS), enable standardized initial assessment and improve patient satisfaction, compared to not applying triage [4–9]. However, triage systems applied in general ED include very few obstetric determinants and are not adapted to the physiological changes and disease spectrum specific to pregnancy and the postpartum period. They are therefore inappropriate for obstetric patients.

Failure to quickly identify and treat critically ill pregnant and recently delivered women seeking acute care has repeatedly led to maternal morbidity and mortality [10–12]. In 2011 Paisley et al. concluded that the lack of Obstetric Triage Systems (OTS) leads to initial assessment of patients based on quick visual evaluation and on time of arrival as well as inconsistency in assessments of patients presenting with similar symptoms [13]. They presented the first OTS, the Florida Hospital Ob Triage Acuity Tool, and emphasized that a triage level was to be assigned before any other examinations were undertaken [13].

Subsequently, other OTS have been developed. Prior to the development of GOTS the Obstetric Triage Acuity Scale (OTAS) [4, 5] and the Maternal Fetal Triage Index (MFTI) were introduced [14, 15]. “Both systems have sufficient interrater reliability. Validity was established with construct and content validity respectively, for OTAS assessing higher levels of admission rates and resources utilization in higher acuity levels, and for MFTI during the development [5, 6, 16]. However, the OTAS did initially not include vital sign parameters to assess the patient’s acuity, an important factor known to strengthen the assessment [17, 18]. MFTI differs significantly from the predominantly used non-obstetric triage system in Sweden. For both systems, evidence of external validity is yet to be as so presented [19, 20]. As with other triage systems, these OTS are adapted to the circumstances, such as local guidelines and previous non-obstetric triage systems, in which they were developed. Furthermore, the systems lacked recommendations for initial management, which would enable staff with limited or no experience in triaging and managing obstetric patients to use the OTS in the general ED setting. Shortly after the development of GOTS, two additional OTS were presented: the obstetric and gynecological amendments to the Swiss Emergency Triage Scale (SETS) and the Birmingham Symptom-specific Obstetric Triage System (BSOTS) was presented [21, 22].

In 2014 Angelini et al. established a Best Practice for obstetric triage, underlining the need for a validated and reliable obstetric triage tool and the importance of teamwork. The obstetric triage process should consist of a 10–20-min initial assessment of both mother and fetus by a nurse or midwife [7]. A 2017 committee opinion by the American College of Obstetricians and Gynecologists urges hospital-based obstetric units to collaborate with EDs to establish guidelines for triage of pregnant women [23].

Sweden has a standardized, free of charge, prenatal care system with more than 99% of pregnant women attending regular appointments. Healthy women with suspected uncomplicated pregnancies are usually handled by midwifes but MDs/obstetricians are consulted and/or responsible for antenatal care in case of intercurrent diseases and pregnancy complications. Proximity to a delivery unit varies greatly related to place of residence. Except for the Stockholm area, there is only one obstetric department in each city/region. Depending on both location of and organization within the different hospitals, pregnant or newly delivered women in need of acute care are assessed in general EDs, in obstetric triage units or in maternity (delivery) units. Staff in these units have varying experience in managing obstetric patients seeking acute care. No department of obstetrics applied obstetric triage prior to this study and no OTS built or adapted to the Scandinavian context was available. The ratio between acute prenatal visits and overall birth volumes ranges from 1.2 to 1.5 [13]. With the Swedish annual birthrate at approximately 115,000, this corresponds to 135,000–170,000 patients being assessed annually with suboptimal routines. There is thus a need for an OTS adapted to the Scandinavian context in both obstetric units as well as general EDs, in order to improve acute care for obstetric patients.

## Methods

The aim of this study was to describe the development, implementation and initial evaluation of an OTS*.*

In 2016 the Department of Obstetrics at Sahlgrenska University Hospital, Gothenburg, Sweden, planned to introduce obstetric triage, due to concerns about patient safety. Sahlgrenska University Hospital is a tertiary referral hospital and the Department of Obstetrics is the largest obstetric unit in Sweden, with approximately 10,000 births per year. Its outpatient unit provides acute care to patients from gestational week 19 until 12 weeks postpartum. There are also scheduled visits for obstetric patients requiring the university hospital’s competence. Routine prenatal care is provided at prenatal clinics under other management and located elsewhere. Women requiring acute care during the first half of pregnancy attend Sahlgrenska University Hospital’s Department of Gynecology.

Prior to April, 2017, most patients with acute symptoms called a coordinator midwife, accessible 24 h daily, before attending. The coordinator referred the patient either to the general ED, the delivery ward, her ordinary prenatal clinic or the outpatient clinic.

**Fig. 1:**
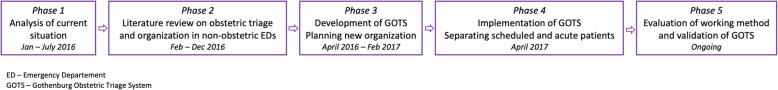
Flowchart of the process to improve acute care of obstetric patients

The development of the OTS took place in five different phasis, see Fig. 1.

### Phase 1. Survey of the demands for a Swedish obstetric triage system

A multidisciplinary team, including obstetricians, midwives, auxiliary nurses and administrative staff, was assembled in order to assess the causes for patients seeking acute care at the obstetric outpatient unit as well as to map the pathway of these patient visits. This multidisciplinary mapping, together with repeated Strengths, Weaknesses, Opportunities and Threats (SWOT) analyses [24], enabled identification of areas with deficiencies in patient safety from a clinical point of view. In addition, a retrospective analysis of 380 consecutive patients’ records concerning unplanned visits during two weeks was performed to identify the most common causes for seeking care. Patient identification data was extracted from the administrative registration system Elvis *(Insieme Consulting AB, Sweden),* for all patients visits. Medical documentation related to the visit was reviewed, identifying the main cause for seeking care.

### Phase 2. Literature review

**Fig. 2:**
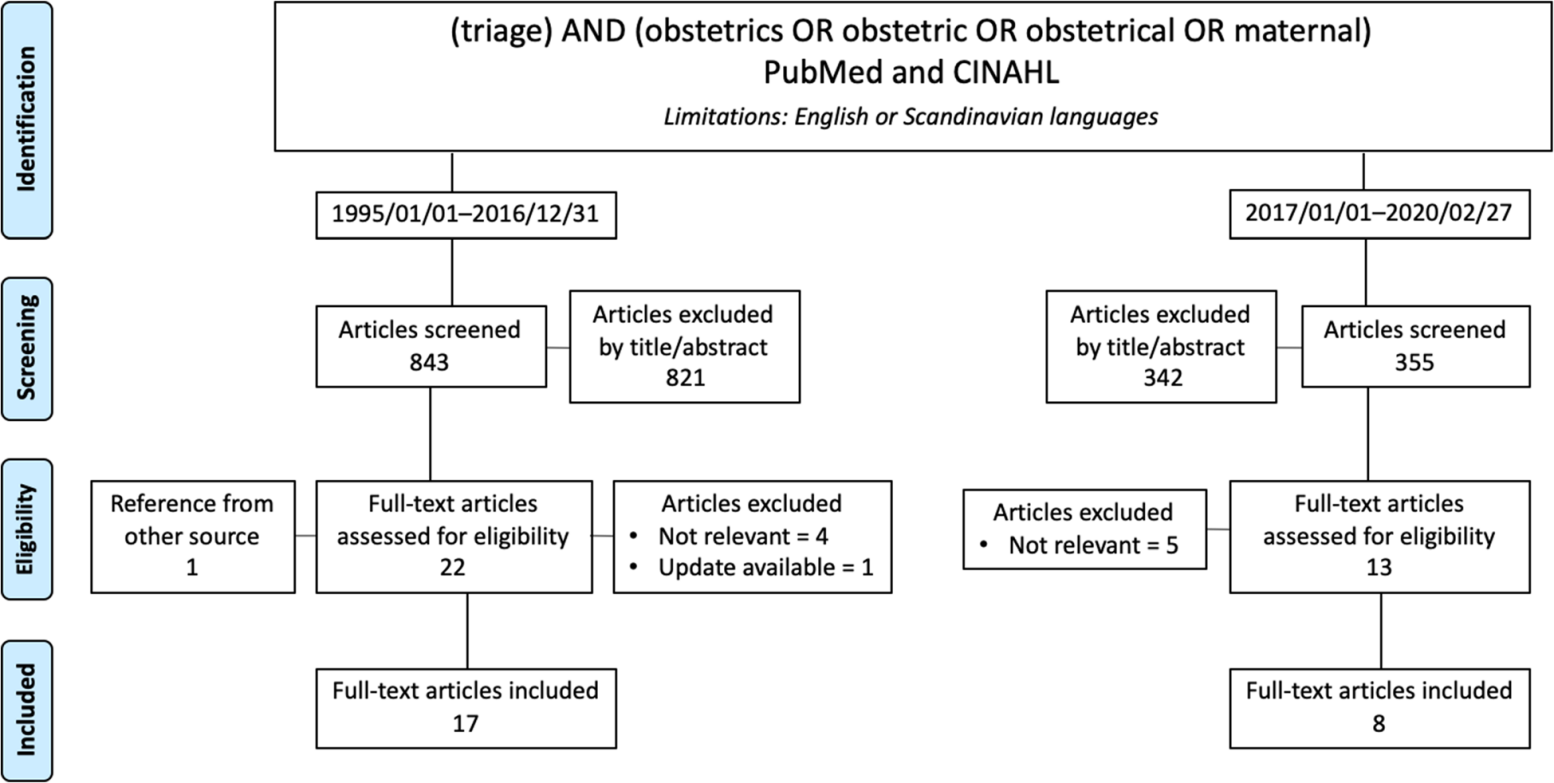
Flowchart of literature review on obstetric triage

A literature search on obstetric triage was conducted with the aid of the medical library at Sahlgrenska University Hospital. The search was performed with the keywords “triage” AND (“obstetrics” OR “obstetric” OR “obstetrical” OR “maternal”) in PubMed and CINAHL limited to publications in English or Scandinavian languages. The first literature search was conducted in 2016 when the project was started comprising the timespan 1995/01/01 to 2016/12/31, and the search was repeated in 2020 to add any new information for this publication (Fig. 2).

### Phase 3. Development of an obstetric triage system

The novel, Swedish OTS was to include both symptoms or chief complaint as well as vital sign parameters adapted to the obstetric patient’s physiology. Furthermore, it was to be designed in accordance with national guidelines and preferably resemble the predominant non-obstetric triage system in Swedish EDs, in order to facilitate implementation in both obstetric and non-obstetric EDs. The OTS was also to enhance the structure of care, enabling a better overview of the patients in the waiting area and improvement of patient safety in alignment with the Best Practice [7]. Furthermore, the changes were to facilitate a structured evaluation of clinical management and implementation was to be accomplished with unaltered or improved patient and staff satisfaction.

A first draft of the Gothenburg Obstetric Triage System (GOTS) was produced, based on knowledge attained from previous obstetric and non-obstetric triage systems. The most common reasons to attend were the foundation for the chief complaint algorithms. Levels of acuity and reference levels for vital sign parameters were determined according to local guidelines, which were in turn based on previous research on normal vital signs in pregnancy [6, 25, 26]. The draft was repeatedly evaluated by the multidisciplinary team, allowing for structural changes if the chief complaint algorithms or the associated acuity chart were found to be clinically inappropriate.

### Phase 4. Implementation

When consensus on the OTS and acuity chart was reached the system was implemented into clinical praxis, with recurring evaluations and discussion in the staff group. To support the implementation, guidelines for the new working method were written. Training and information were provided to both medical and administrative staff at regular workplace meetings, allowing for questions and reflections to be addressed. A flowchart of the implementation process provided up-to-date information and the implementation date was set, after which members of the multidisciplinary team were scheduled to work at the unit daily for the first month.

In addition to the development of GOTS, administrative, organizational and facility changes were made to enable the working method of obstetric triage. These included introduction of a time registration system as well as assigning staff (midwives, auxiliary nurses and obstetricians) to shifts devoted exclusively to triage and subsequent care of acute patients. All midwives at the unit were trained to perform triage, but they also rotated to other work stations and shifts. A triage room was appropriately equipped, e.g. with triage algorithms, equipment for assessing vital parameters, Doppler ultrasound for detecting fetal heartbeat, a stretcher and a computer. Scheduled and unscheduled patients were separated into different waiting areas. Furthermore, posters in the waiting area provided information regarding GOTS.

### Phase 5. Initial evaluation

Patient satisfaction was continuously monitored by an electronic questionnaire available in Swedish, English and Arabic. The questionnaire is administered postpartum in clinical routine to all women who delivered at Sahlgrenska University Hospital *(**supplementary**).* Women answered on a five-level response scale regarding satisfaction with treatment, participation in decision-making and accessibility of unit. In addition, they were able to write free comments. Results were presented as a composite, weighted outcome. Staffs’ satisfaction was continually, verbally evaluated on regular staff meetings. LOS and admission rates according to acuity levels were monitored by an administrative time registration system, Elvis/Power BI Report, with monthly reports.

## Results

### Phases 1 and 2

The predominant causes for contacting the coordinator or attending the unit were reduced fetal movements, vaginal bleeding, abdominal pain, signs of hypertensive disorder including preeclampsia, signs of prenatal or postpartum infection, premature contractions, contractions at term, suspected rupture of membranes and signs of mastitis. These conditions, together with severe conditions such as trauma towards the pregnant abdomen,headache/neurological symptoms, postpartum hemorrhage, chest pain, dyspnea and signs of venous thromboembolism constituted the foundation for the chief complaint algorithms.

Mapping the care process revealed the following areas requiring improvement:

- The care process was unstructured and the staff felt uncertain, to some extent, when assessing patients.
- Patients were registered at the reception by staff with no medical training and then placed in a waiting area without medical assessment, although the midwives did keep an eye on them while they waited.
- Scheduled and acute patients shared the same waiting area. No midwives or auxiliary nurses were designated to assess and care exclusively for the acute patients. The obstetricians handled a mixture of scheduled and acute patients.
- Patients that looked ill or expressed that they were in pain were seen first, but this prioritization did not always correspond to the actual acuity level.
- Assessment was dependent on the midwives’ experience and could hence differ from day to day.
- The management structure did not enable adequate evaluation of the organization, due to lack of pre-determined benchmarks and time registration. Planning for adequate staffing was thus difficult.
- The organization led to near misses, where patients not assessed in time might have suffered severe complications.

Results of the literature review are shown in Fig. 2 and Table 1 (Additional file 1). The literature review and perusal of studies on general emergency care revealed two factors, key to improving care [4, 9, 27] - Triage, including a triage area and an OTS as well as separating patients seeking acute care from scheduled patients. These two key factors corresponded well with the deficiencies identified at the unit. Regrettably, the literature review did not provide guidance in which OTS to use since comparative studies do not exist. Furthermore, there is no consensus in outcome measurement of internal validity and external validity of existing systems is unknown.

### Phase 3. Development of the Gothenburg obstetric triage system

#### The Gothenburg obstetric triage system

**Fig. 3:**
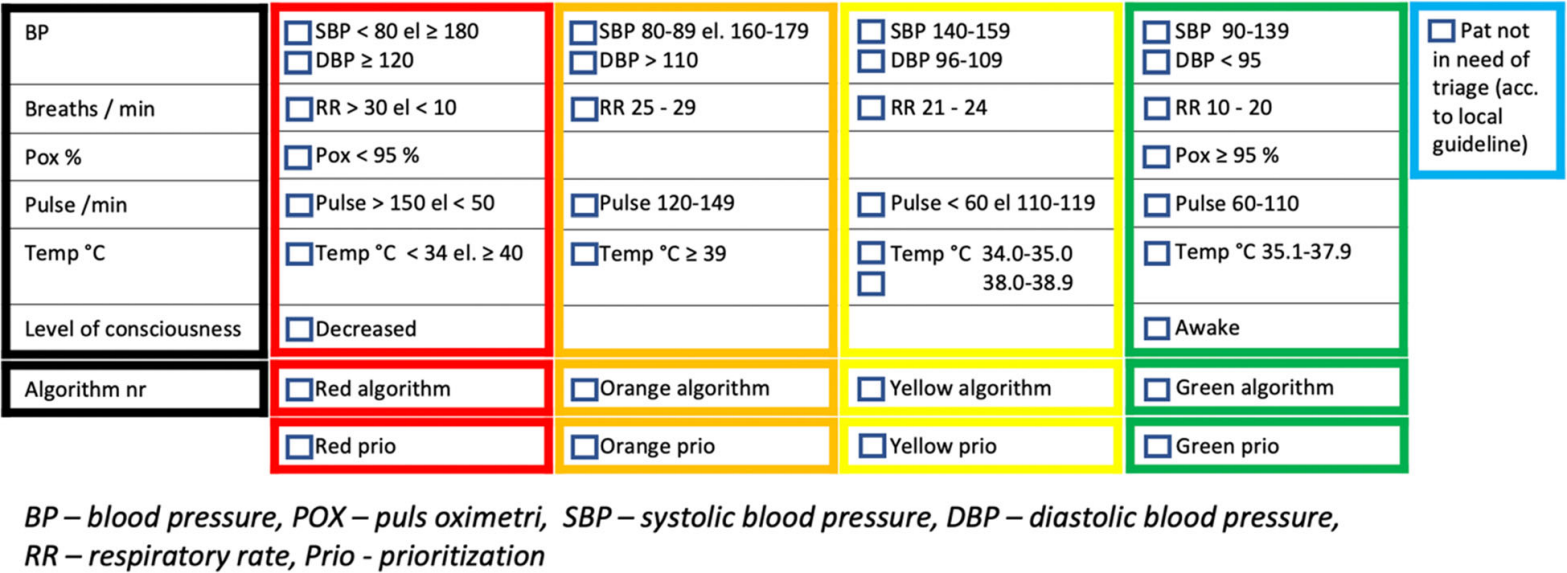
Pregnancy-adapted reference levels for vital sign parameters

**Fig. 4:**
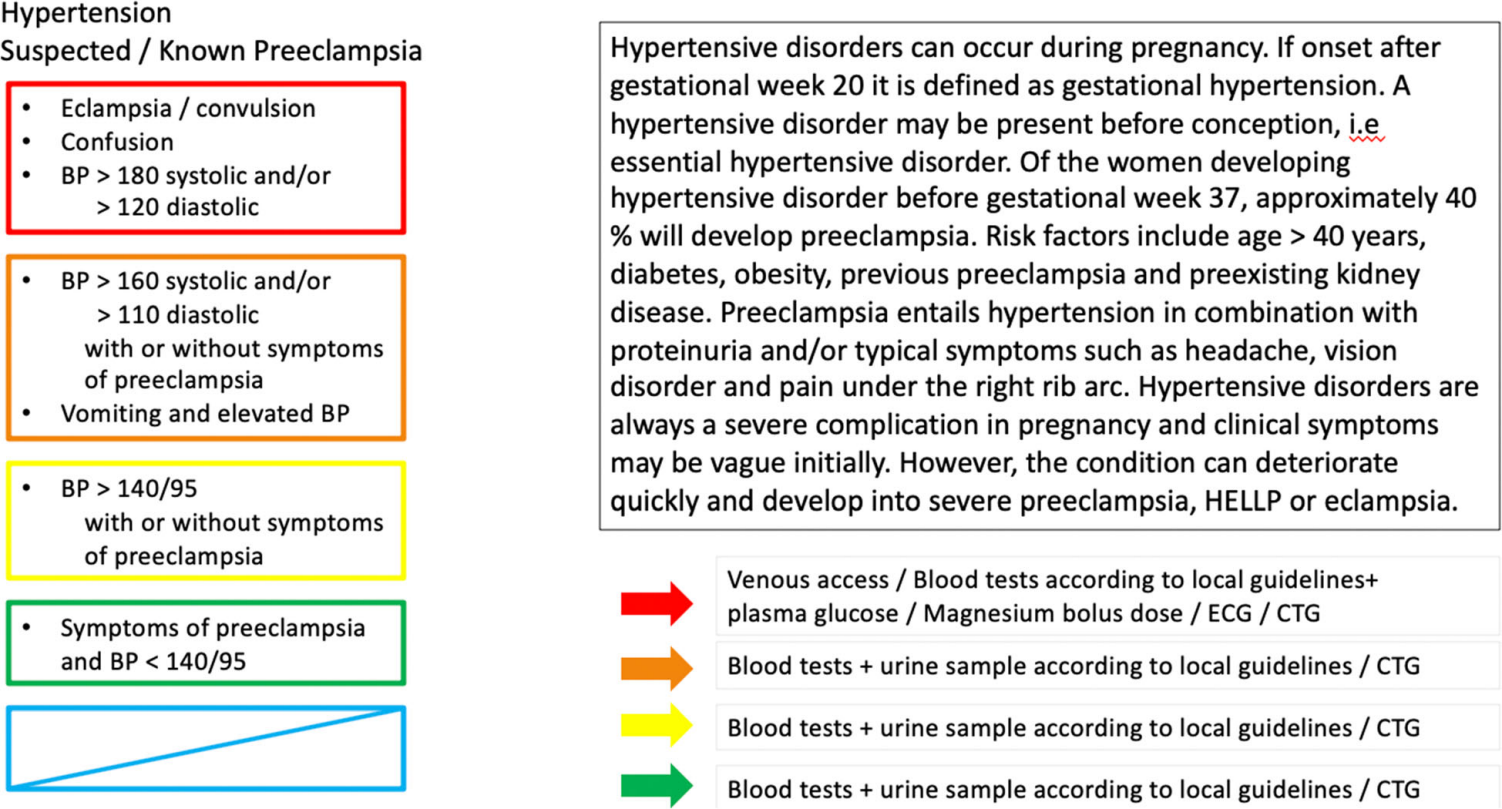
Example of a chief complaint algorithm. *Hypertensive disorder or suspected/known preeclampsia*

The GOTS is a five-tier triage scale, structurally similar to RETTS, that includes structured reevaluation during the waiting time. The GOTS acuity level assessment is based both on vital signs and 14 chief complaint algorithms (Fig. 3). The reference levels for vital signs are adapted to the physiological changes in pregnancy (Fig.4. The algorithms comprise contractions, suspected rupture of membranes, vaginal bleeding, reduced fetal movements, suspected hypertensive disorder, neurological symptoms, abdominal/back pain, trauma, postpartum hemorrhage, signs of prenatal or postpartum infection, chest pain/breathing problems, suspected thromboembolism, hyperemesis and suspected mastitis. The algorithms include information on both obstetric and non-obstetric possible causes for the symptoms and on initial treatment and investigations, such as laboratory tests. The system also entails an acuity form for documentation.

#### Workflow based on the Gothenburg obstetric triage system

The patient is seen by a midwife in triage and the chief complaint and vital sign parameters as well as fetal heartbeat are assessed and registered on the acuity form *(*Fig. 4*)*. One of five acuity levels, ranging from red (immediate evaluation) through orange (urgent, evaluation within 20 min) to yellow-green-blue (non-urgent), is selected. The thresholds for wait time in the different acuity levels were modelled after RETTS [18], the predominately used non-obstetric triage system in Sweden at the time when GOTS was developed. If two divergent acuity levels are indicated by the chief complaint and the vital signs, the higher level is chosen. Yellow to blue levels entail low risk of critical illness and the patient can wait, with the goal of evaluation set to 60, 120 and 240 min respectively. After triage, further examinations are conducted in accordance with the level of acuity. In all patients, the primary evaluation and treatment is conducted by a midwife and about 30–40% of the patients are managed without involvement of an obstetrician. If the patient shows signs of circulatory and/or respiratory failure or presents with symptoms of stroke she is directly referred to the general ED. Treatment for stabilization of the patient is preformed to ensure safe transfer.

### Phase 4. Implementation

#### Organizational effects

**Fig. 5:**
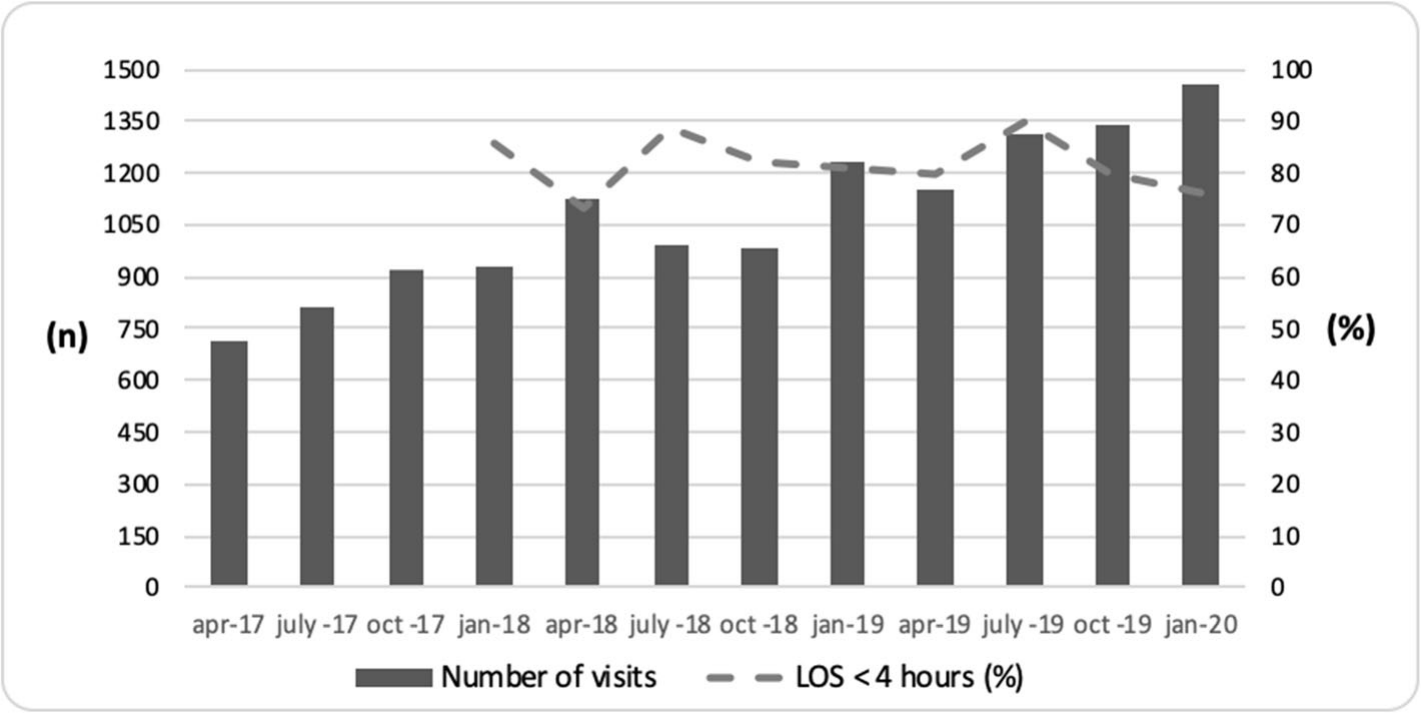
Monthly visits and LOS < 4 h. *Visits at the Obstetric Emergency Department at Sahlgrenska University Hospital, Gothenburg, Sweden, April 2017 to January 2020*

**Fig. 6:**
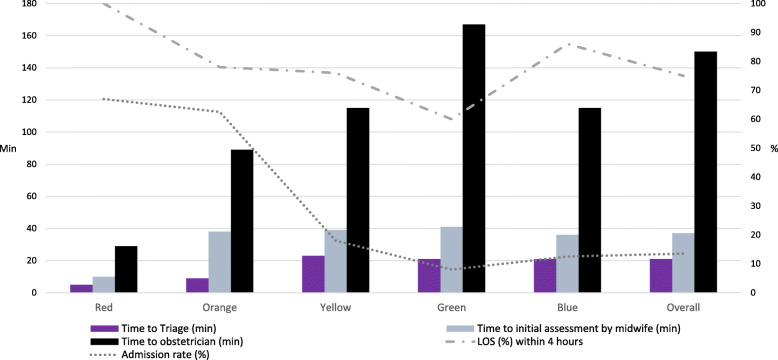
Time to triage, time to assessment, length of stay (LOS) and admission rates. *Time registrations stratified according to acuity level – falling urgency from red to blue. Obstetric Emergency Department, Feb-March 2020, 2486 visits*

The change in working method has enabled the staff to better evaluate its effectiveness, for instance changes in total LOS (Fig. 5), waiting times (Fig. 6) and total obstetric triage volumes per hour in real time. Approximately 80% of all patients have a total LOS < 4 h, maintaining this level even though number of visits have increased by almost 40% since the registration started. This facilitates more effective staff allocation and process planning. Outcome variables have been set in accordance with those in general emergency care at Sahlgrenska University Hospital [28].

### Phase 5. Initial evaluation

Initial evaluation of the system indicates success in identifying patients requiring admission into appropriate acuity levels (Fig. 6). The most critically ill (red) patients are seen within 10 min. However, orange-level patients are not always assessed within the target of 20 min. Time to assessment at this level is equivalent to that of the non-urgent acuity levels (Fig. 6). Time to assessment by an obstetrician is proportional to the acuity level. We were not able to compare LOS before and after GOTS was introduced as LOS was not measured in a structured way prior to the implementation of an OTS. However, since 2018, the percentage of patients with LOS under 4 h has remained at a constant level, i.e. around 80% despite the fact that the number of patients seeking acute care has increased by approximately 40% during the study period (Fig. 5).

#### Patient satisfaction

Questionnaire response rate was approximately 70%, with little variation over several years. Patient satisfaction increased from around 80% before introduction of GOTS to 86% (Table 1).

**Table 1:** Patient satisfaction with the care at the obstetric emergency department from 2015 to 2019

***Patient satisfaction (%)***	2015	2016	2017	2018	2019
Composite, weighted outcome	80	80	80	82	86

Patient satisfaction as assessed by questionnaire before and after introduction of GOTS in April 2017. Results are presented as a composite, weighted outcome consisting of satisfaction with treatment, participation in decision-making and accessibility of unit according to a five-level response scale.

#### Staff satisfaction

Staff members report a valid sensation of increased security, manifested by the knowledge of “who is waiting in the waiting area” and structure, manifested by a simplification in knowing in which order to assess the patients and directions in initial management, after implementation. The staff also experience less perceived stress and communication between professions is perceived as objective, clearer and more direct, aligning with previous research [29]. The unit has developed an emergency care profile and is now classified as one of Sahlgrenska University Hospital’s EDs. Staff is much better prepared for severely ill patients and all professions have a better understanding of initial management and treatment.

## Discussion

We present the first Swedish obstetric triage system, the GOTS, its development, implementation and initial evaluation. GOTS enables prioritization of obstetric patients seeking acute care based on clinical urgency and can be used in both obstetric and non-obstetric EDs. Obstetric triage based on the GOTS, has been successfully implemented at the largest Department of Obstetrics in Scandinavia, the Sahlgrenska University Hospital. The implementation has provided the staff with an improved medical overview of the patients and patient flow and enabled the unit to monitor emergency care in a structured way. GOTS successfully identifies patients requiring admission. Implementation came along with increased patient and staff satisfaction.

Previous studies have used the effect on LOS – length of stay – as a primary outcome to measure the effectiveness of triage, showing varying results [4, 30]. This outcome measurement is relevant from an organizational point of view and for patient satisfaction [30, 31] and has traditionally been used since it is easy to register and evaluate. Nevertheless, evaluating LOS is not without difficulty. Since 2018, the percentage of patients with LOS under 4 h has remained at a constant level, i.e. around 80% despite the fact that the number of patients seeking acute care has increased during the study period. We firmly believe that without GOTS, the obstetric ED would not have been able to uphold this performance. It must, however, also be emphasized that LOS is dependent on other factors within the department, such as the discharge rate which enables admission of new patients from the ED [30]. All department units are interrelated and the workload at any unit affects the others.

The challenge in evaluating LOS is also evident in the evaluation of waiting time and admission rate in the blue GOTS category, that includes patients with suspected rupture of membranes and patients presenting for scheduled visits. The latter occur, despite the explicit goal of separating emergency and planned visits, since the number of available semi-acute appointments is as yet insufficient at Sahlgrenska University Hospital. Primiparous patients with confirmed rupture of membranes are, according to local guidelines, followed for three days by the obstetric ED before induction and are triaged to be seen before acute patients classified as green. The same applies for patients that should have had a scheduled semi-acute appointment. This is the reason for blue patients having higher admission rates and a lower LOS than green patients (Fig. 6). Hence, it is important to find complementing outcome variables in order to assess effectiveness and functionality, with focus on aspects such as patient safety and workplace environment [7, 31–33].

The unit has failed to achieve an overall time to triage of < 10 min, mainly due to limited facilities. With only one room allocated for triage and an approximately 10-min long assessments, more rapid turnover is impossible. Interestingly, time to triage, i.e. before any type of medical assessment, correlates with acuity level. This is partly due to some of the severely ill patients arriving by ambulance, resulting in immediate triage. Even the administrative staff has obtained a sense of emergency care and will now notify midwives more quickly if they suspect that the patient is severely ill. While time to initial midwife assessment, after triage, is almost equal for all other patients than red (Fig. 6), longer waiting time to obstetrician assessment was associated with lower acuity. This waiting time includes 10–60 min of CTG in the majority of cases.

This study shows that GOTS enables rapid identification and assessment of patients with the highest level of acuity and that acuity level correlates well with admission rates, suggesting good validity. More comprehensive studies on GOTS validity are currently performed. The system presents as a good foundation for improved and adapted triage of obstetric patients even in general EDs. Even though obstetric patients will best be taken care of in a designated obstetric unit, some conditions such as severe trauma or acute chest pain in the pregnant and newly delivered woman may be better treated in a general ED. As many geographical areas lack designated obstetric units, the obstetric patient might seek care at any unit providing emergency and urgent care. Hence, coordination and communication regarding these patients as well as introducing reliable triage systems including obstetric determinants is of great importance [23].

It can be discussed whether to develop another OTS when there are already existing OTS. At the time of GOTS development (2016) only three OTS were available– Florida Hospital Ob Triage Acuity Tool, MFTI and OTAS. To the best of our knowledge, the Florida Hospital Ob Triage Acuity Tool has not been evaluated on reliability nor validity. Studies on OTAS and MFTI have shown sufficient inter-rater reliability. However, OTAS, with some similarities to the predominantly used non-obstetric triage system in Sweden, did not include vital sign parameters for assessment and the MFTI, developed in accordance to the non-obstetric triage system ESI, differs significantly from the predominantly used non-obstetric triage system in Sweden, which might impair the implementation as well as complicate the collaboration with non-obstetric EDs. Alongside the fact that there is no consensus on which system to apply, lacking of scientific support for their external validity [34], the internal validity of the triage systems being questioned [19, 20] and the systems not offering sufficient support to staff with scares or no experience in triaging and managing obstetric patients, it was decided to develop an OTS suitable for the Swedish context.

### Strengths and limitations

GOTS is the first OTS in Sweden. Development by a multidisciplinary team working clinically with acute obstetric patients is a strength as it ensures that the OTS is adapted to the local needs and prerequisites. Basing assessments on both chief complaint algorithms and vital signs with pregnancy-adapted reference levels is another strength. However, this study also has limitations. Due to inadequate time registration prior to implementation of GOTS, comparisons regarding patient flow through the obstetric ED are not possible. This is currently addressed in implementation studies of GOTS at other Swedish obstetric departments. As previously mentioned, it can be questioned whether LOS is an adequate measurement of acute care quality because of the risk of disturbing factors. However, time to triage and time to further examination is of great importance for patient safety and is more directly related to the urgency of the patient’s presenting symptom. In this study, more in-depth evaluation of staff satisfaction with the working method was difficult since the staff has been involved in the continuous development and implementation and hence, might be biased. This subject is now under evaluation in a mixed-method study, implementing GOTS in other obstetric departments.

Both reliability and validity studies, addressing patient safety, are currently performed and studies on patient flow and the staff’s experience of working with obstetric triage have been initiated. With the rising need for structured and cost-effective care, and the aim to decrease maternal morbidity and mortality rates, obstetric triage will become increasingly important.

## Conclusions

The GOTS is the first OTS developed in and for Sweden and implementation has led to improved management of obstetric patients seeking acute care. Patients are now prioritized according to level of acuity and the time to assessment and treatment of severely ill patients can be followed in a structured way. Both patients and staff express improved satisfaction with obstetric triage. Further research to improve both the triage system and working method has been initiated.

## Supplementary Information

**Additional file 1: Table S1.** Summary of semi-structured literature review on obstetric (Ob) triage

**Additional file 2.** Questionnaire for evaluation of patient satisfaction. Questions are extracted from a more extensive questionnaire, used routinely as a quality evaluation by the unit

## Data Availability

The datasets analyzed for LOS, time to triage and admission rates, during the current study are not publicly available due to the risk of identifying patients seeking emergency care at the unit but are available from the author on reasonable request.

## Abbreviations

ED: emergency department
GOTS: Gothenburg Obstetric Triage System
LOS: length of stay
OTS: obstetric triage system
SU: Sahlgrenska University Hospital
