# Supplementary material for: Improving assessment of acute obstetric patients – introducing a Swedish obstetric triage system
Source: BMC Health Serv Res. 2021 Nov 6;21:1207. doi: 10.1186/s12913-021-07210-9 (PMC8572438; doi:10.1186/s12913-021-07210-9)
Supplement: Supplementary file 2 — Additional file 2. Questionnaire for evaluation of patient satisfaction. Questions are extracted from a more extensive questionnaire, used routinely as a quality evaluation by the unit [file 12913_2021_7210_MOESM2_ESM.docx]

**Supplementary** Questionnaire for evaluation of patient satisfaction. Questions are extracted from a more extensive questionnaire, used routinely as a quality evaluation by the unit.

| 1. | How old are you? |  |  | | |  | | |  | |  |  |  |
| --- | --- | --- | --- | --- | --- | --- | --- | --- | --- | --- | --- | --- | --- |
|  | Younger than 20 years |  |  | | |  | | |  | |  |  |  |
|  | 20 – 30 years |  |  | | |  | | |  | |  |  |  |
|  | 31 – 40 years |  |  | | |  | | |  | |  |  |  |
|  | Older than 40 years |  |  | | |  | | |  | |  |  |  |
|  |  |  |  | | |  | | |  | |  |  |  |
| 2. | **Is it your first or subsequent birth?** |  |  | | |  | | |  | |  |  |  |
|  | First birth |  |  | | |  | | |  | |  |  |  |
|  | Subsequent |  |  | | |  | | |  | |  |  |  |
|  |  |  |  | | |  | | |  | |  |  |  |
| 3. | **Based on your visit to emergency room for pregnant and newly delivered,**  **how satisfied are you with...** | Very satisfied | Satisfied | | Less Satisfied | | | Dissatisfied | | Don’t know/no opinion | | | |
|  | Accessibility |  |  | |  | | |  | |  | | | |
|  | Treatment during the visit |  |  | |  | | |  | |  | | | |
|  | Participation in the decisions taken |  |  | |  | | |  | |  | | | |
|  |  |  |  | |  | | |  | |  | | | |
| 4. | **Do you have any comments, tips or thoughts you would like to pass on to the staff at the emergency room?** | |  |  | | |  | | |  | | |  |
|  |  | |  |  | | |  | | |  | | |  |
|  |  | |  |  | | |  | | |  | | |  |
|  |  | |  |  | | |  | | |  | | |  |
|  |  | |  |  | | |  | | |  | | |  |
|  |  | |  |  | | |  | | |  | | |  |
|  |  | |  |  | | |  | | |  | | |  |
